# Integration of histone modification-based risk signature with drug sensitivity analysis reveals novel therapeutic strategies for lower-grade glioma

**DOI:** 10.3389/fphar.2024.1523779

**Published:** 2025-01-13

**Authors:** Jingyuan Wang, Shuai Yan

**Affiliations:** ^1^ Department of Neurological Surgery, The First Affiliated Hospital of China Medical University, Shenyang, China; ^2^ Department of Neurological Function Examination, Affiliated Hospital of Hebei University, Baoding, China

**Keywords:** lower-grade glioma, histone modification, risk signature, drug sensitivity, prognosis, machine learning

## Abstract

**Background:**

Lower-grade glioma (LGG) exhibits significant heterogeneity in clinical outcomes, and current prognostic markers have limited predictive value. Despite the growing recognition of histone modifications in tumor progression, their role in LGG remains poorly understood. This study aimed to develop a histone modification-based risk signature and investigate its relationship with drug sensitivity to guide personalized treatment strategies.

**Methods:**

We performed single-cell RNA sequencing analysis on LGG samples (n = 4) to characterize histone modification patterns. Through integrative analysis of TCGA-LGG (n = 513) and CGGA datasets (n = 693 and n = 325), we constructed a histone modification-related risk signature (HMRS) using machine learning approaches. The model's performance was validated in multiple independent cohorts. We further conducted comprehensive analyses of molecular mechanisms, immune microenvironment, and drug sensitivity associated with the risk stratification.

**Results:**

We identified distinct histone modification patterns across five major cell populations in LGG and developed a robust 20-gene HMRS from 129 candidate genes that effectively stratified patients into high- and low-risk groups with significantly different survival outcomes (training set: AUC = 0.77, 0.73, and 0.71 for 1-, 3-, and 5-year survival; *P* < 0.001). Integration of HMRS with clinical features further improved prognostic accuracy (C-index >0.70). High-risk tumors showed activation of TGF-β and IL6-JAK-STAT3 signaling pathways, and distinct mutation profiles including TP53 (63% vs 28%), IDH1 (68% vs 85%), and ATRX (46% vs 20%) mutations. The high-risk group demonstrated significantly elevated immune and stromal scores (*P* < 0.001), with distinct patterns of immune cell infiltration, particularly in memory CD4^+^ T cells (*P* < 0.001) and CD8^+^ T cells (*P* = 0.001). Drug sensitivity analysis revealed significant differential responses to six therapeutic agents including Temozolomide and targeted drugs (*P* < 0.05).

**Conclusion:**

Our study establishes a novel histone modification-based prognostic model that not only accurately predicts LGG patient outcomes but also reveals potential therapeutic targets. The identified associations between risk stratification and drug sensitivity provide valuable insights for personalized treatment strategies. This integrated approach offers a promising framework for improving LGG patient care through molecular-based risk assessment and treatment selection.

## 1 Introduction

Low-grade glioma (LGG) is a primary brain tumor originating from glial cells (Weller et al., 2024), accounting for 7.6% of all primary brain tumors and 31.8% of gliomas. The median overall survival ranges from 5.6 to 13.3 years, depending on tumor histopathological characteristics, molecular phenotype, and growth rate (Li et al., 2021a). The optimal treatment strategy for LGG remains controversial, with current approaches including surgical resection, radiotherapy, chemotherapy, targeted therapy, and immunotherapy (van den Bent et al., 2023). Although LGG patients have slightly better survival rates than those with high-grade (WHO grade III or IV) gliomas (Aiman et al., 2024), the infiltrative nature of gliomas makes LGG prone to drug resistance and recurrence after treatment (Fukuya et al., 2019), with potential progression to high-grade gliomas, significantly shortening survival time (Tom et al., 2019). Following local dissemination, LGG tumor cells exhibit high heterogeneity (Nicholson and Fine, 2021), leading to greater variations in patient survival rates and times. This cellular diversity and heterogeneity in LGG are considered primary factors in tumor recurrence and malignant transformation (Ye et al., 2024; Gittleman et al., 2019). Therefore, understanding the cellular mechanisms underlying LGG development is crucial for clarifying its progression and developing new effective therapeutic targets to extend patient survival.

Histone modification is a crucial epigenetic regulatory mechanism encompassing various forms, including methylation, acetylation, phosphorylation, adenylation, ubiquitination, and ADP-ribosylation (Millán-Zambrano et al., 2022). With rapid advances in molecular biology, the World Health Organization substantially updated its diagnostic criteria for LGG in 2021, transitioning from traditional histological diagnosis to an integrated diagnostic system incorporating molecular markers (Figarella-Branger et al., 2022). This shift is prominently reflected in the WHO CNS5 classification system, which establishes IDH mutation and 1p/19q codeletion status as core molecular markers for adult-type diffuse low-grade glioma classification, fully reflecting the molecular heterogeneity of LGG.

Currently, epigenetic alterations (including histone methylation, DNA methylation, and histone acetylation) are increasingly being applied in brain tumor research (Han et al., 2024). While histone modifications have been extensively studied in high-grade gliomas (HGG), such as proteomics combined with other multi-omics revealing the central role of PTPN11 signaling in high-grade gliomas (Lowe et al., 2019a), and histone H3 mutations promoting diffuse glioma development through chromatin dysregulation (Lowe et al., 2019b), related research in LGG remains relatively scarce. Given the common cellular origins between LGG and HGG (Network et al., 2015), these findings may hold significant implications for LGG as well. Considering that tumor cell heterogeneity is a key factor in LGG recurrence and malignant transformation, conducting more extensive and comprehensive studies on histone modifications is crucial. This not only helps deepen our understanding of LGG’s molecular pathogenesis but also provides new insights for developing personalized treatment strategies for highly heterogeneous LGG.

In our study, we conducted a series of complex bioinformatics analyses, utilizing high-throughput sequencing and proteomics technologies to monitor genome-wide histone modification dynamics, while employing diverse machine learning frameworks and big data to perform comprehensive systematic analysis and identification of histone modifications and related multi-omics features in LGG. By integrating genomics, transcriptomics, and proteomics data to establish machine learning models, we comprehensively revealed key molecules and pathways controlling LGG development and treatment response. This cross-omics integrated analysis approach not only deepens our understanding of LGG epigenetic regulation but also identifies new biomarkers and potential therapeutic targets, potentially providing new directions for LGG treatment research.

## 2 Materials and methods

**FIGURE 1:**
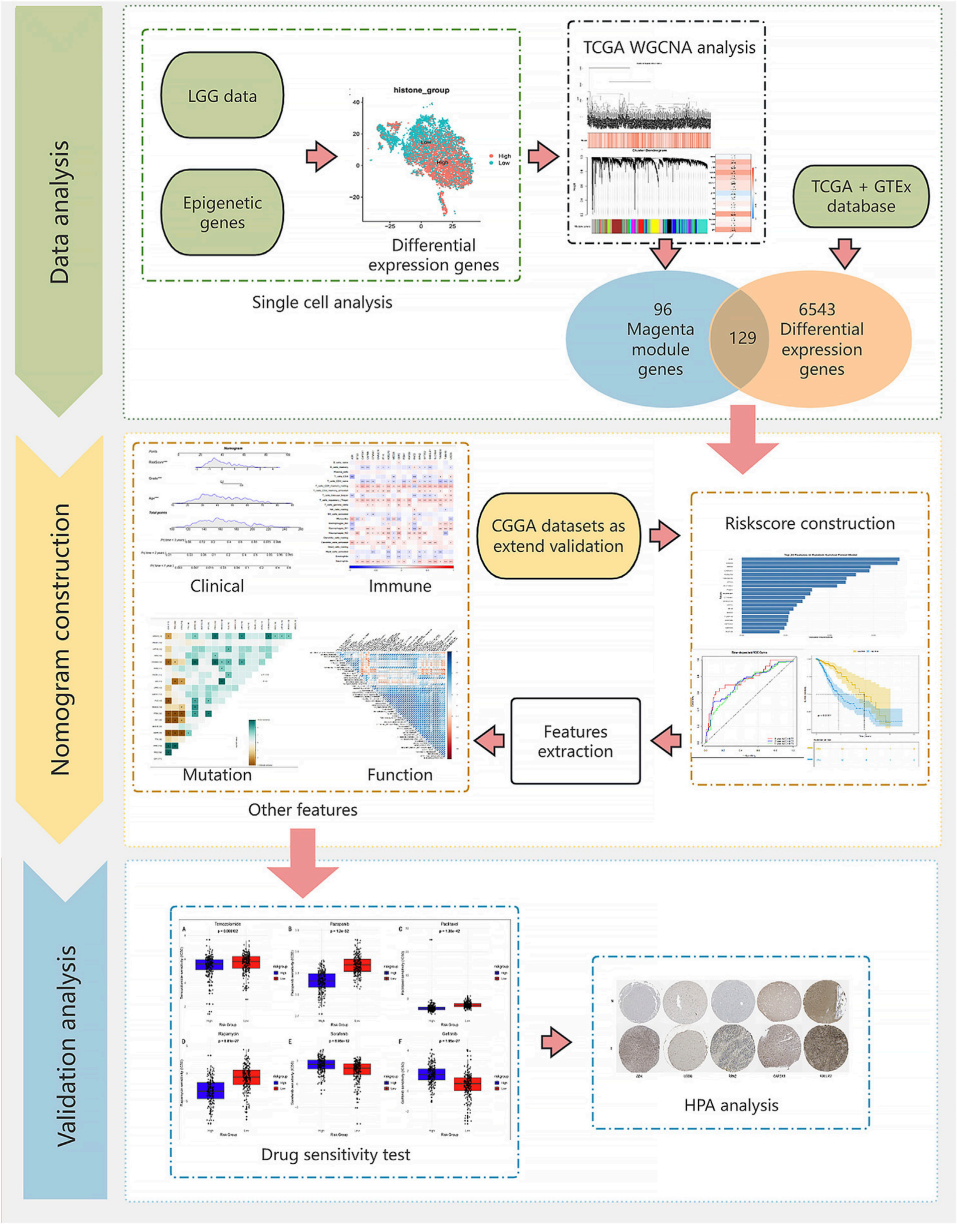
Flow chart.

The research workflow is shown in Figure 1.

### 2.1 Data source

This study integrated multiple independent datasets to construct and validate a prognostic model for low-grade glioma. Initially, we established a histone modification gene set based on Füllgrabe et al. (2011)’s research and the GeneCards database (https://www.genecards.org/) (relevance score >20). Subsequently, single-cell transcriptome data from 4 LGG samples (GSE182109) were obtained from the GEO database (https://www.ncbi.nlm.nih.gov/geo/) for further single-cell analysis. TCGA-LGG (513 samples) and GTEx (2,642 normal control samples) datasets were merged after batch effect removal to calculate differentially expressed genes in LGG. Additionally, TCGA-LGG served as the training set, while two independent cohorts from the Chinese Glioma Genome Atlas (CGGA) database (https://www.cgga.org.cn/), containing 693 and 325 GBM samples respectively with clinical and survival information, were used as external validation sets.

### 2.2 Single cell analysis

#### 2.2.1 Data preprocessing and quality control

Single-cell RNA sequencing data were analyzed using Seurat (v5.0.0) (Abdelfattah et al., 2022). Initially, raw data in 10X Genomics format were processed and preliminarily filtered, requiring each gene to be expressed in at least three cells and each cell to express at least 250 genes. Subsequently, the expression proportions of mitochondrial genes (prefixed with MT-) and ribosomal genes [prefixed with RP (SL)] were calculated for each cell. To ensure data quality, we filtered out the following cells: 1) cells expressing <100 or >7,500 genes; 2) cells with mitochondrial gene expression proportion >25%; 3) cells with total RNA counts <1,000.

#### 2.2.2 Data integration and annotation

Filtered data were normalized using the “LogNormalize” method (Luo et al., 2019) with a scale factor of 10,000. The “vst” method was used to select 2,000 highly variable genes for subsequent analysis. To eliminate batch effects, the Harmony algorithm was employed to integrate data from multiple samples (Petegrosso et al., 2020; Korsunsky et al., 2019). Subsequently, PCA dimensionality reduction was performed, selecting the top 30 principal components for further analysis. Using these principal components, UMAP and t-SNE dimensionality reduction visualizations were performed separately. Cell clustering was conducted using a graph-based clustering algorithm (Petegrosso et al., 2020), with the optimal resolution parameter of 0.2 determined through the clustree package. Cell types were automatically annotated using the SingleR package (Aran et al., 2019) in conjunction with the Human Primary Cell Atlas database (https://www.humancellatlas.org/) and scMayomap package (Yang et al., 2023). Additionally, manual verification was performed based on characteristic gene expression (Hassn Mesrati and Behrooz, 2020), including: OLIG2 and MBP (oligodendrocyte markers), CD44 and SOX2 (tumor cell markers), PDGFRB (pericyte marker), FOLR2, AIF1, and CD68 (macrophage markers), CD8A, CD3E, and CCL5 (T cell markers).

#### 2.2.3 Histone score analysis

Based on the predefined histone gene set, the ssGSEA algorithm was used to calculate histone scores for each cell (Chen et al., 2022). For LGG cell subgroups, cells were divided into high and low expression groups based on the median histone score, and differential expression analysis was performed (logFC threshold of 0.5, minimum expression proportion of 0.35) to identify functional pathways associated with histone expression.

### 2.3 Weighted gene co‐expression network analysis

WGCNA analysis was conducted based on the previously obtained histone-related differentially expressed gene set (Xu et al., 2023) to reveal gene co-expression relationships and their associations with phenotypes. During data preprocessing, gene expression data from the TCGA-LGG dataset were normalized, and quality was ensured by removing genes with zero standard deviation and samples with missing values. Subsequently, the goodSamplesGenes function was used for quality assessment, and outlier samples were detected using hierarchical clustering (Grabski et al., 2023). In network construction, the optimal soft threshold power = 5 was determined by analyzing the scale-free topology fit index and average connectivity under different soft thresholds. The blockwiseModules function was used to construct the co-expression network, setting the minimum module size to 50 genes, module merging similarity threshold to 0.15, and using unsigned network type. Through calculating Module Eigengenes (MEs) (Han et al., 2019), we analyzed the correlation between modules and histone scores, using Pearson correlation coefficients to evaluate module-trait relationships, and assessed correlation significance using Student’s t-test. The correlation intensity between modules and phenotypic features was visualized through heatmaps. Finally, in modules significantly correlated with histone scores, the biological significance was validated by analyzing the relationship between Module Membership and Gene Significance, with scatter plots demonstrating their positive correlation, further confirming these modules’ central role in the histone regulatory network.

### 2.4 Feature genes selection

Differential expression analysis was performed using the TCGA-LGG dataset and GTEx dataset through DESeq2, with selection criteria of absolute logFC >1 and *p*-value <0.05. Feature genes were selected by intersecting the differentially expressed genes with module genes obtained from WGCNA.

### 2.5 Machine learning based prognosis signature construction

To construct a reliable prognostic prediction model, this study used the TCGA-LGG dataset as the training set and CGGA325 and CGGA693 datasets as independent validation sets. Initially, all datasets underwent standardization and feature space consistency was ensured, with missing values eliminated through strict data quality control. During model construction, we systematically evaluated multiple machine learning algorithms and their combinations, including Random Survival Forest (RSF), Elastic Net (Enet), Stepwise Cox regression (StepCox), CoxBoost, Partial Least Squares Regression (plsRcox), Principal Component Regression (SuperPC), Gradient Boosting Machine (GBM), Survival Support Vector Machine (survival-SVM), Ridge regression, and Lasso regression as base models. To enhance prediction performance, we explored various combinations of these base models, such as RSF with CoxBoost and Lasso with GBM combinations. For the Elastic Net model, performance was optimized by adjusting the α parameter (0.1–0.9); for stepwise Cox regression, forward, backward, and bidirectional feature selection methods were employed. Model evaluation used C-index (Song et al., 2022) as the primary evaluation metric, assessing predictive ability and generalization performance through comprehensive performance on training and two independent validation sets. Finally, model performance across different datasets was visualized through heatmaps, and models were ranked based on average C-index values from validation sets to select the final prognostic prediction tool with optimal predictive efficacy and stability.

### 2.6 Optimal model performance validation and risk score construction

Based on the comprehensive evaluation results of machine learning models, the optimal prediction model underwent thorough performance validation and risk score system construction. Specifically, through feature importance analysis, 20 key features contributing most significantly to prognosis prediction were identified from the original features. Based on the model’s predictions, a risk score system was established, dividing patients into high and low-risk groups using the median score as the threshold, and Kaplan-Meier survival analysis was performed to observe survival differences between risk groups. Additionally, to evaluate the model’s time-dependent predictive ability, time-dependent ROC curves (Obuchowski and Bullen, 2018) and corresponding AUC values were calculated for 1-year, 3-year, and 5-year predictions.

### 2.7 Prognostic value analysis of clinical features and risk scores

The prognostic value of clinical features and risk scores was evaluated through systematic survival analysis (Schober and Vetter, 2018). Initially, univariate Cox regression analysis assessed the impact of clinical features including age, gender, tumor type, grade, and risk score on patient prognosis. Subsequently, multivariate Cox regression analysis identified independent prognostic factors. Based on significant independent prognostic factors, an integrated nomogram prediction model (Gittleman et al., 2019) was constructed, and its prediction accuracy was verified through calibration curves for 1-year, 3-year, and 5-year survival predictions. Time-dependent C-index analysis was used to compare the predictive performance between the nomogram model and individual predictive factors, while decision curve analysis (DCA) evaluated the clinical net benefit of the model at different decision thresholds to validate this prediction tool’s value in clinical practice.

### 2.8 Enrichment analysis

This study explored molecular pathway differences between high and low risk score groups through systematic functional enrichment analysis (Canzler and Hackermüller, 2020). First, Gene Set Enrichment Analysis (GSEA) evaluated significantly enriched Hallmark pathways in the high-risk group. Subsequently, Gene Set Variation Analysis (GSVA) scored all samples, and limma differential analysis identified pathways with significantly different activities between high and low-risk groups. To verify the clinical relevance of key pathways, patients were divided into high and low pathway activity groups based on GSVA scores, with Kaplan-Meier survival analysis evaluating prognostic differences, and Cox proportional hazards regression model calculating hazard ratios (HR) and their 95% confidence intervals. For significantly correlated pathways, forest plots were generated to visualize their prognostic value. Finally, survival curves were verified for the six most significant pathways, comprehensively assessing these pathways’ potential roles in glioma development and progression.

### 2.9 Mutation analysis and heterogeneity assessment

This study explored the association between tumor heterogeneity and risk scores through comprehensive analysis of somatic mutation data from the TCGA-LGG cohort. First, the Mutant-Allele Tumor Heterogeneity (MATH) score (Timar and Kashofer, 2020) was used to quantify tumor heterogeneity levels for each sample, comparing differences between high and low-risk groups. Distribution characteristics were visualized through violin plots, with statistical significance assessed using the Wilcoxon rank-sum test. Subsequently, patients were divided into high and low heterogeneity groups based on the median MATH score, with Kaplan-Meier survival analysis evaluating the relationship between tumor heterogeneity and prognosis. Further combining MATH scores with risk scores, patients were classified into four subgroups (high MATH/high risk, high MATH/low risk, low MATH/high risk, low MATH/low risk) to explore the joint predictive value of both indicators. Finally, maftools was used to analyze mutation characteristics of high and low-risk groups, with waterfall plots displaying distribution characteristics of top 20 mutated genes, while co-occurrence and mutual exclusivity analysis (Zang et al., 2023) revealed interaction patterns among key driver genes.

### 2.10 Immune analysis

This study conducted systematic analysis of the LGG tumor immune microenvironment using multiple algorithms. Initially, the ESTIMATE algorithm (Tennant et al., 2022) calculated stromal scores, immune scores, and overall scores for each sample, comparing differences between high and low-risk groups. Subsequently, immune-related pathways were scored using ssGSEA, with heatmaps visualizing differential patterns of immune pathway activity between risk groups. Further, the CIBERSORT algorithm (Chen et al., 2018) was employed to deconvolute the infiltration proportions of 22 immune cell types, with violin plots showing immune cell composition differences between high and low-risk groups. Additionally, ssGSEA analysis was performed using 28 immune cell characteristic gene sets, with box plots clearly displaying abundance differences of various immune cell types between risk groups. Finally, correlation analysis explored relationships between key gene expression and immune cell infiltration, as well as associations between risk scores and immune cell infiltration levels. Significant correlation patterns were displayed through heatmaps and correlation scatter plots, revealing potential connections between the risk score model and tumor immune microenvironment.

### 2.11 Drug sensitivity analysis

Systematic drug sensitivity prediction analysis was performed on the TCGA-LGG cohort using the pRRophetic package (Yan et al., 2022). Initially, half maximal inhibitory concentration (IC50) values (Sebaugh, 2011) were predicted for all available drugs based on drug response data from the Cancer Genome Project (CGP) database. For each drug, drug sensitivity differences between high and low-risk groups were compared, with statistical significance assessed using the Wilcoxon rank-sum test. For drugs showing significant differences (*P* < 0.05), box plots were used to visually display drug sensitivity distribution characteristics across different risk groups.

### 2.12 Human protein atlas validation

Based on previous analysis results, we selected the top five key genes with the highest weights for expression validation in the HPA database (https://www.proteinatlas.org/). Through immunohistochemical staining images, protein expression levels and distribution patterns of these key genes were visually demonstrated in normal brain tissue and glioma tissues of different grades. The validation results from the HPA database not only confirmed the differential expression characteristics of these genes in glioma development at the protein level but also provided histological evidence for understanding their potential roles in tumor progression.

### 2.13 Pan-cancer analysis

To comprehensively visualize the pan-cancer analysis results, we constructed a stratified forest plot integrating survival analysis outcomes across 29 cancer types, organized by eight major organ systems (Breast and Gynecologic System, Respiratory System, Digestive System, Urinary System, Nervous System, Endocrine System, Male Reproductive System, Hematologic System, and Others). In the forest plot, dot sizes represent -log10 (*p*-value), error bars indicate 95% confidence intervals, and color-coding displays risk levels (red for high-risk groups, cyan for low-risk groups, and gray for non-significant differences). A horizontal dashed line (HR = 1) serves as a reference, and hazard ratios are presented on a logarithmic scale to better illustrate the relative magnitude of risk differences.

## 3 Results

### 3.1 Single-cell data reveals differential distribution of histone modifications

**FIGURE 2:**
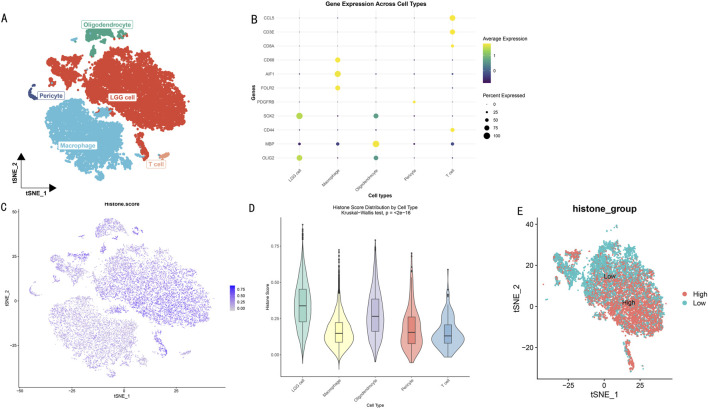
Histone Modification Lineage Analysis Based on Single-Cell Transcriptomics. **(A)** t-SNE dimensionality reduction showing spatial distribution of LGG cell subgroups. Different colors represent different cell subgroups. **(B)** Heatmap of cell subgroup-specific marker gene expression profiles. Rows represent genes, columns represent cells. **(C)** t-SNE plot showing distribution characteristics of histone modification scores across different cell subgroups. Color intensity indicates modification levels. **(D)** Box plot analysis of histone modification scores for five cell subgroups. **(E)** Bidirectional clustering analysis based on histone modification scores. t-SNE projection showing distribution patterns of high-score (red) and low-score (blue) cells.

Through dimensionality reduction clustering analysis and cell type annotation of LGG single-cell data (Figure 2A), we successfully identified five major cell subpopulations: SOX2 and OLIG2-expressing LGG tumor cells, CD68, AIF1, and FOLR2-expressing macrophages, MBP-expressing oligodendrocytes, PDGFRB-expressing pericytes, and CCL5, CD3E, CD8A, and CD44-expressing T cells. To validate the accuracy of cell type annotation, we constructed a dot plot displaying the expression patterns of marker genes for each cell type (Figure 2B).

To deeply explore the heterogeneity of histone modification levels among different cell types, we calculated and visualized histone modification scores for each cell subgroup (Figure 2C). Results showed significant differences in histone modification levels among different cell subgroups. Further statistical analysis (Figure 2D) revealed that LGG tumor cells and oligodendrocytes exhibited higher histone modification levels (scores >0.25). Based on the overall cellular histone modification levels (HMs), we separated LGG cells into high HMs and low HMs groups (Figure 2E). Differential expression analysis identified 5,278 differentially expressed genes (|logFC|>1, *p* < 0.05), with 3,638 genes upregulated in the high HMs group and 1,640 genes upregulated in the low HMs group.

### 3.2 HMs-related gene network analysis

**FIGURE 3:**
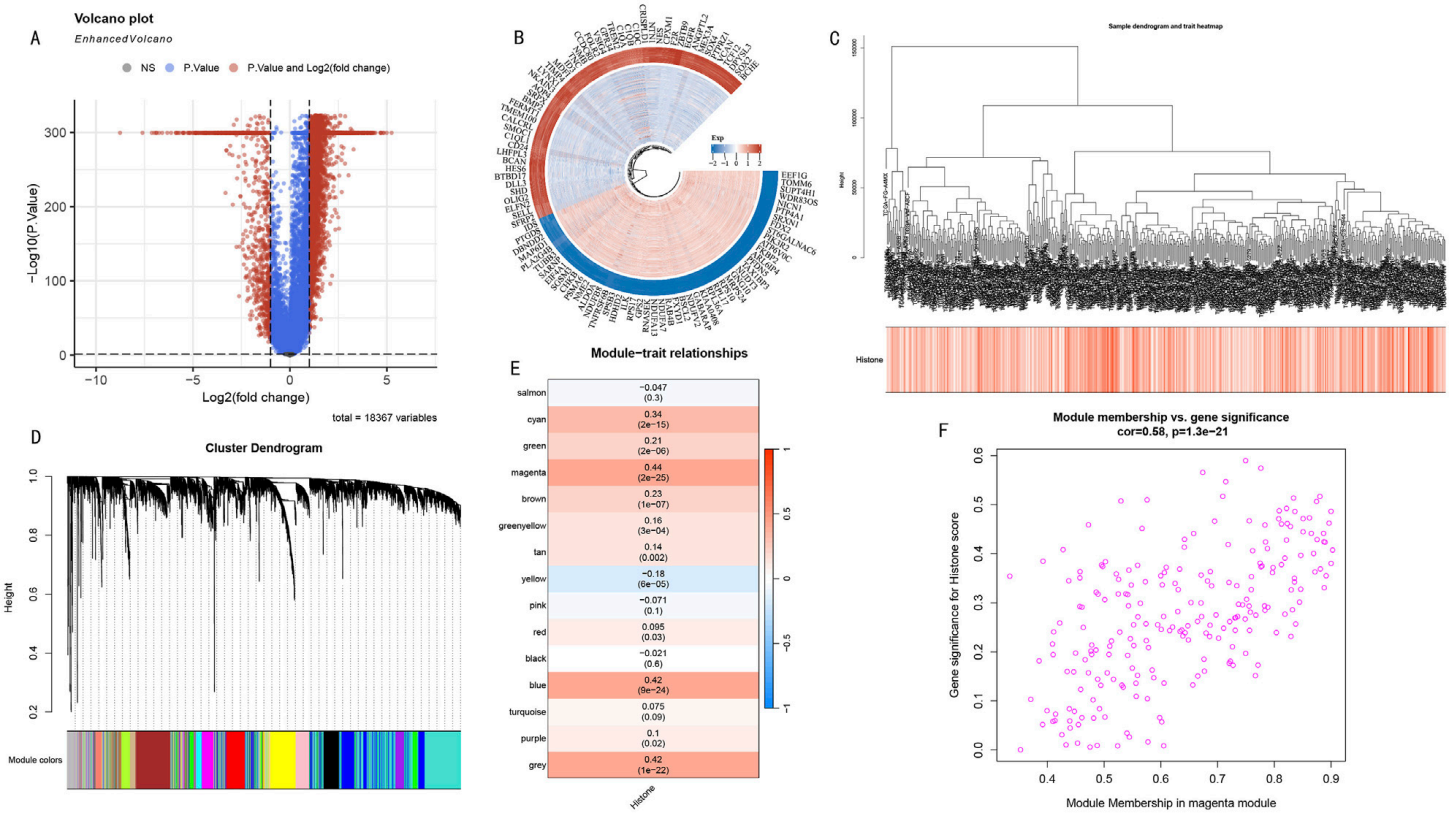
WGCNA Network Analysis Reveals Key Gene Modules in Histone Modification Regulation. **(A)** Volcano plot distribution of differentially expressed genes. Red and blue dots represent significantly upregulated and downregulated genes in the high HMs group, respectively. **(B)** Circular plot of top 50 most significantly up- and downregulated genes. Inner to outer rings show gene names, expression change direction, and statistical significance. **(C)** Hierarchical clustering dendrogram of differentially expressed genes. Upper part shows hierarchical clustering relationships among genes, lower heatmap shows distribution characteristics of sample HMs scores. Color intensity represents score levels. **(D)** WGCNA module identification results. Dendrogram shows gene clustering relationships, bottom colored bands represent 15 functional modules identified by dynamic tree-cutting algorithm. **(E)** Module-trait correlation heatmap. Each row represents a co-expression module, values and color intensity indicate Pearson correlation coefficients with histone modification scores. Magenta module shows strongest positive correlation (cor = 0.44, *P* < 0.005). **(F)** GS-MM scatter plot analysis of magenta module. X-axis: Module Membership; Y-axis: Gene Significance. Distribution trend (cor = 0.58, *P* < 1e-21) validates this module’s core position in histone modification regulatory network.

Differential expression analysis revealed significant transcriptomic differences between high and low HMs groups. Volcano plot analysis showed numerous genes with significant differential expression (Figure 3A), suggesting these genes may participate in the histone modification regulatory network. To deeply analyze key regulatory genes, we visualized the top 50 up- and downregulated genes with the most significant differences in a circular plot (Figure 3B). To systematically identify co-expression modules related to histone modifications, we performed WGCNA analysis on differentially expressed genes. The hierarchical clustering dendrogram displayed gene co-expression relationships, while the bottom heatmap reflected HMs score variation patterns among samples (Figure 3C). Based on the dynamic tree-cutting algorithm, we ultimately identified 15 functional modules with significant co-expression characteristics (Figure 3D). Module-trait correlation analysis indicated that the magenta module (225 genes) showed the strongest positive correlation with histone modification scores (cor = 0.44, *P* < 0.005) (Figure 3E). Further module membership analysis revealed that genes in the magenta module showed significant positive correlation between Gene Significance (GS) and Module Membership (MM) (cor = 0.58, P < 1e-21) (Figure 3F), strongly suggesting this module plays a core role in the histone modification regulatory network.

### 3.3 Construction of prognostic model using feature genes

**FIGURE 4:**
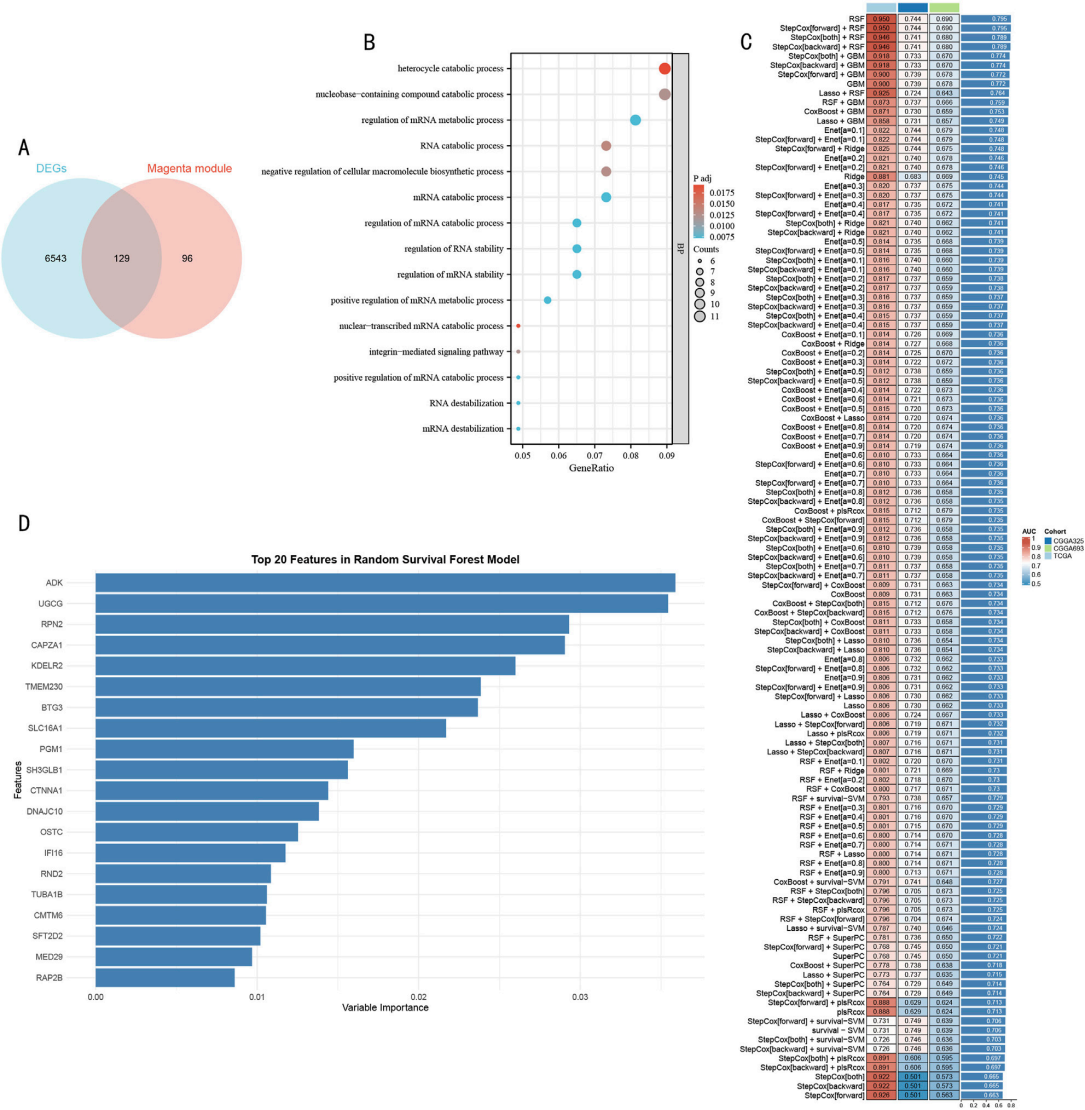
Systematic Identification and Model Construction of Histone Modification-Related Prognostic Markers **(A)** Venn diagram analysis of LGG DEGs and magenta module genes. **(B)** GO functional enrichment dot plot of LGG-HMRgenes. X-axis represents gene ratio, dot size represents number of enriched genes, color intensity represents statistical significance [-log10 (P-value)]. **(C)** Machine learning model performance evaluation heatmap. Rows represent different algorithms, columns represent validation datasets. Color scale indicates C-index values (red indicates higher prediction accuracy, blue indicates lower prediction accuracy). **(D)** Importance ranking plot of Top 20 feature genes identified by RSF model. X-axis represents feature importance scores, Y-axis represents gene symbols. Bar length reflects each gene’s contribution to prognostic prediction.

Through integrative transcriptomic analysis of TCGA-LGG and GTEx datasets, we initially identified 6,672 LGG-related differentially expressed genes (DEGs), including 5,798 upregulated and 874 downregulated genes. Intersection analysis of these DEGs with previously determined magenta module genes yielded 129 LGG-specific histone modification-related genes (LGG-HMRgenes) (Figure 4A). Functional enrichment analysis revealed these LGG-HMRgenes were significantly enriched in pathways including heterocycle catabolic process, nucleobase-containing compound catabolic process, RNA catabolic process, and negative regulation of cellular macromolecule biosynthetic process (Figure 4B, *P* < 0.05). These results emphasize the regulatory role of histone modifications in LGG development and provide theoretical basis for developing targeted therapeutic strategies.

To construct a robust prognostic prediction model, we systematically evaluated the predictive performance of 105 machine learning algorithms using 129 HMR genes as feature inputs (Figure 4C). Through comprehensive comparison of C-index performance across validation sets, the Random Survival Forest (RSF) model demonstrated optimal predictive performance. Based on feature importance analysis of the RSF model, we further selected 20 core feature genes with the strongest predictive contributions (Figure 4D).

### 3.4 Multi-center validation and performance assessment of HMRS prognostic model

**FIGURE 5:**
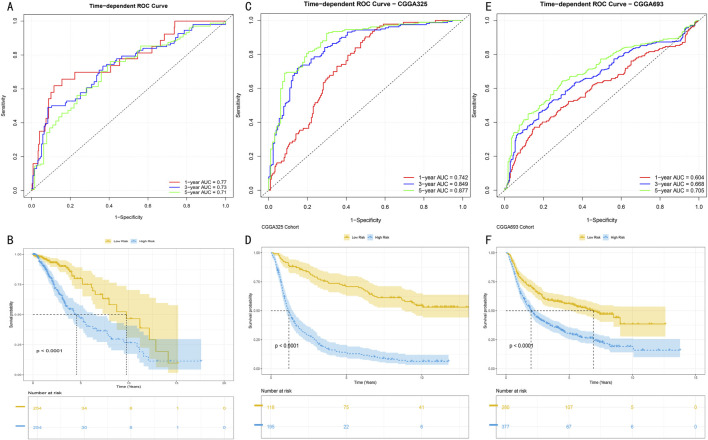
Predictive Performance and External Validation of HMRS Model **(A)** Time-dependent ROC curve analysis in TCGA training set. Red, blue, and green curves represent AUC values for 1-year, 3-year, and 5-year survival predictions. **(B)** Kaplan-Meier survival analysis based on HMRS scores in TCGA cohort. Yellow and blue curves represent high-risk group (n = x) and low-risk group (n = y) respectively. Shaded areas indicate 95% confidence intervals. **(C, E)** Time-dependent ROC curves in CGGA325 and CGGA693 validation sets. AUC values at various time points demonstrate the model’s stable predictive performance. **(D, F)** Survival stratification analysis in validation sets. Separation of survival curves validates the model’s prognostic discrimination ability (log-rank test, *P* < 0.001). Numbers at bottom indicate number at risk at each time point.

To systematically evaluate the time-dependent predictive performance of the HMRS model, we first conducted time-dependent receiver operating characteristic (ROC) analysis in the TCGA-LGG cohort (513 samples). Results showed that the model demonstrated excellent discriminative ability in 1-year, 3-year, and 5-year survival predictions, with corresponding areas under the curve (AUC) reaching 0.77, 0.73, and 0.71 respectively (Figure 5A). After stratifying patients into high and low-risk groups based on the optimal cutoff value, Kaplan-Meier survival analysis revealed significant prognostic differences between the groups (log-rank test, *P* < 0.001) (Figure 5B).

To validate the external applicability of the HMRS model, we conducted validation in two independent CGGA validation cohorts (CGGA325 and CGGA693). In the CGGA325 cohort (325 samples), the model demonstrated time-dependent prediction accuracy comparable to the training set (Figure 5C), with survival stratification differences showing statistical significance (*P* < 0.001) (Figure 5D). These results were further confirmed in the CGGA693 cohort (693 samples) (Figures 5E, F). Multi-center validation results confirmed that the HMRS model possesses robust prognostic prediction capability and broad clinical applicability.

### 3.5 Construction and evaluation of clinical variable-integrated prognostic model

**FIGURE 6:**
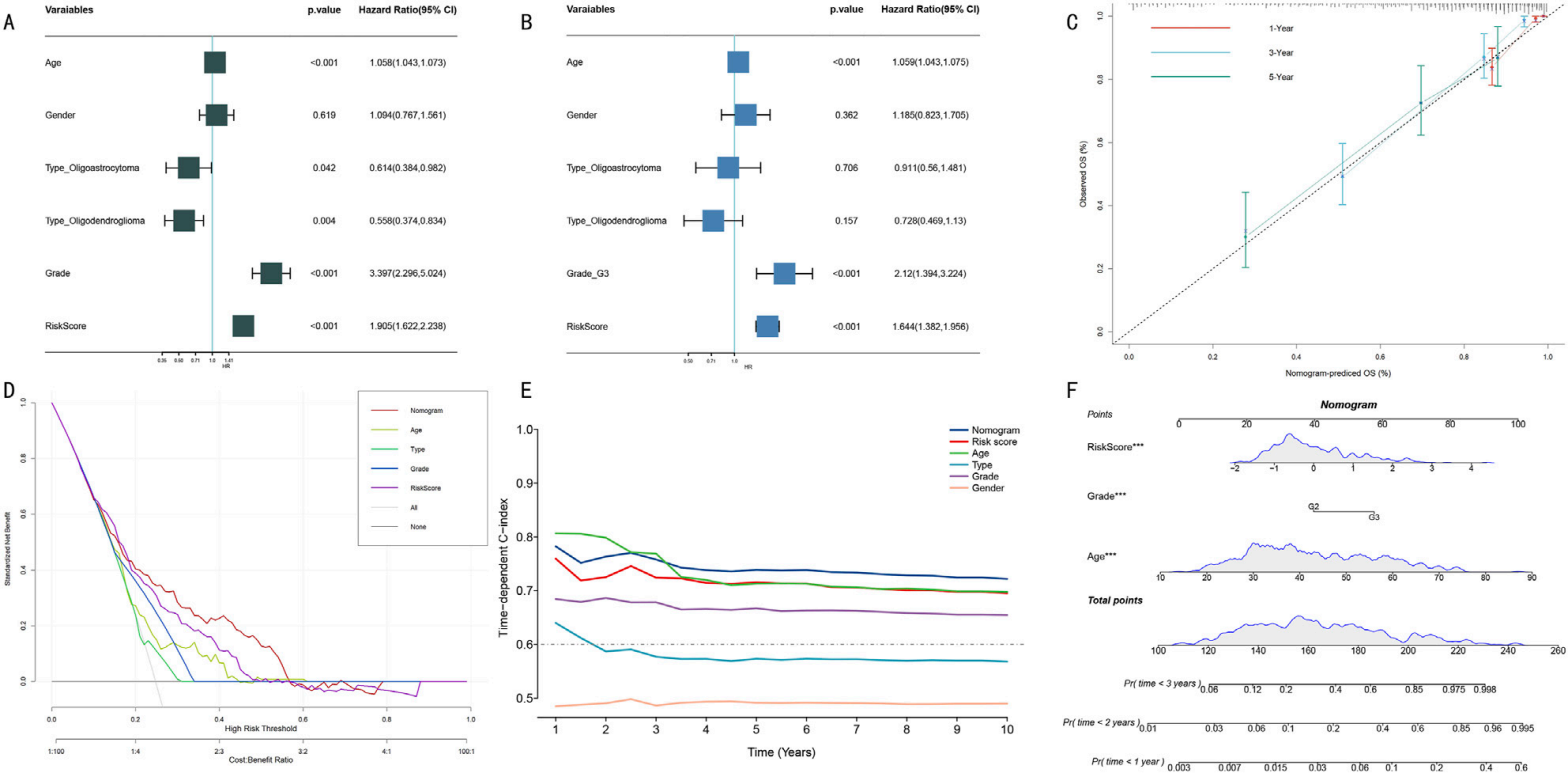
Construction and Performance Evaluation of Integrated Prognostic Prediction Model. **(A)** Forest plot of univariate Cox regression analysis. Shows hazard ratio (HR) and 95% confidence intervals for each clinical feature. **(B)** Forest plot of multivariate Cox regression analysis. Confirms independent prognostic factors (age, WHO grade, HMRS score). **(C)** Calibration curves for nomogram model. Shows consistency between predicted and actual survival probabilities at 1-year (red), 3-year (blue), and 5-year (green). Diagonal line represents perfect prediction. **(D)** DCA. Compares net benefit of different prediction strategies at various risk thresholds. **(E)** Dynamic comparison of time-dependent C-indices. Shows prediction accuracy of nomogram model (red) versus single prognostic factors at different follow-up time points. **(F)** Integrated prognostic prediction nomogram. Includes three independent prognostic factors: HMRS score, WHO grade, and age, for individualized prognosis prediction.

To systematically evaluate the prognostic value of clinical features and HMRS scores, we first conducted Cox proportional hazards regression analysis. Univariate analysis showed that age, WHO grade, and HMRS score were significant prognostic factors (Figure 6A, all *P* < 0.001). Multivariate analysis further confirmed the independent prognostic value of these three factors (Figure 6B). Based on these independent prognostic factors, we constructed an integrated nomogram prediction model. Calibration curve analysis showed that the model demonstrated excellent calibration in 1-year, 3-year, and 5-year survival predictions (Figure 6C).

Clinical decision curve analysis (DCA) indicated that the integrated nomogram model demonstrated greater net benefit compared to single prognostic factors (Figure 6D). Dynamic analysis of time-dependent C-index showed that the nomogram model’s prediction accuracy (C-index >0.70) consistently outperformed single prognostic factors (Figure 6E). Notably, although age performed well in short-term (1–3 years) prediction, its long-term prediction stability was insufficient. Finally, we established a visual nomogram incorporating HMRS score, WHO grade, and age (Figure 6F), providing an intuitive quantitative tool for clinical prognostic assessment.

### 3.6 Systematic functional analysis of HMRS-related molecular mechanisms

**FIGURE 7:**
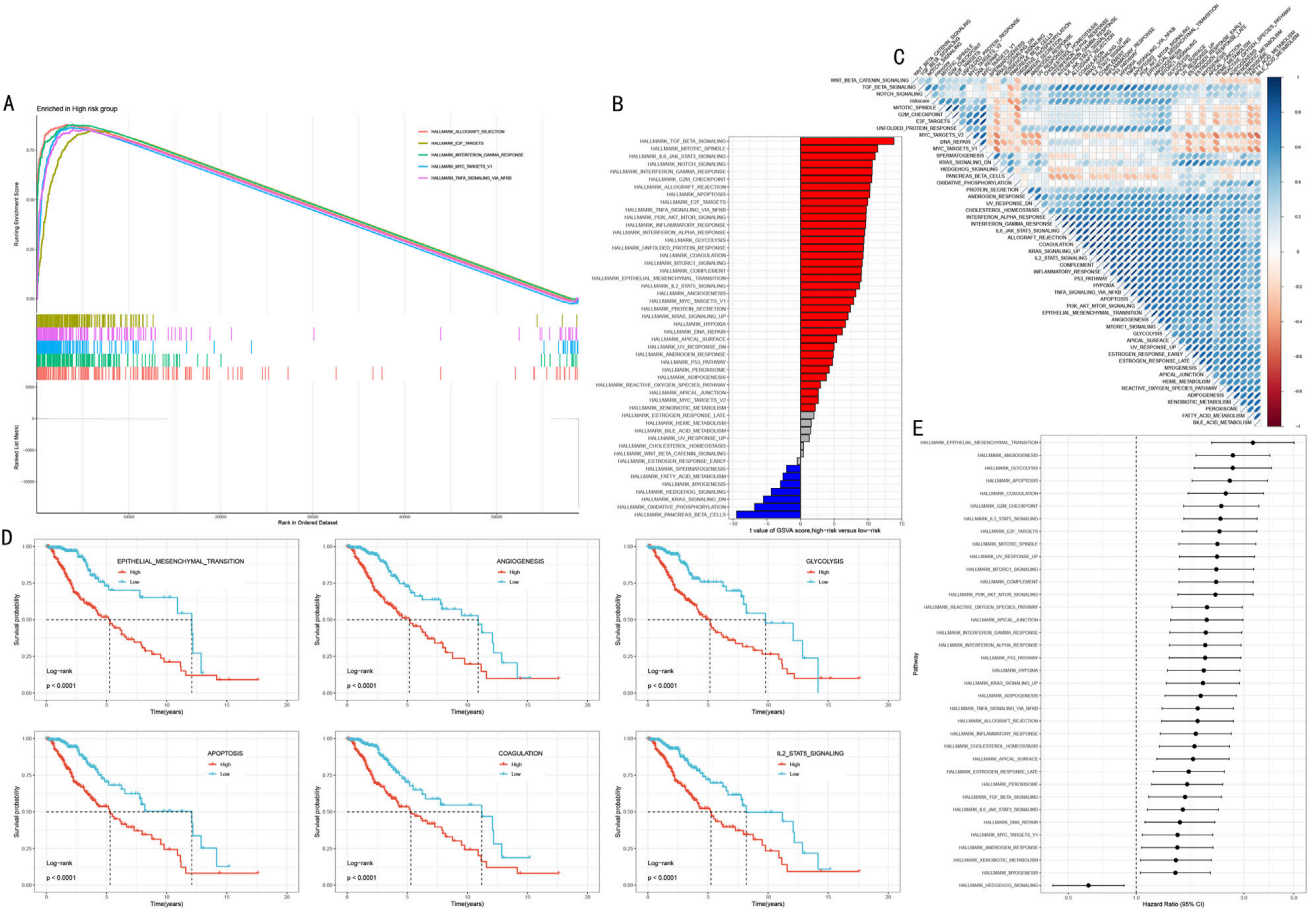
Multi-dimensional Functional Analysis of HMRS-Related Molecular Mechanisms. **(A)** GSEA waterfall plot showing five key pathways significantly enriched in high-risk group. Upper part shows enrichment plots, lower part shows gene expression heatmap. **(B)** Differential pathways revealed by GSVA analysis between high and low-risk groups. Red and blue indicate upregulated pathways in high-risk and low-risk groups respectively. **(C)** Correlation heatmap between HMRS scores and key pathway activities. Red and blue indicate positive and negative correlations respectively. **(D)** Survival analysis of six key pathways. Patients divided into high activity (red line) and low activity (blue line) groups based on pathway activity scores. **(E)** Forest plot of pathway hazard ratios. Shows degree of impact and 95% confidence intervals of each pathway on prognosis.

To elucidate the molecular biological basis of HMRS prognostic stratification, we conducted systematic functional enrichment analysis between high and low-risk groups. GSEA showed that the high-risk group was significantly enriched in multiple cancer-related Hallmark pathways, including Allograft Rejection, E2F Targets, Interferon Gamma Response, MYC Targets V1, and TNFα Signaling via NFκB (Figure 7A, FDR <0.05).

Gene Set Variation Analysis (GSVA) further revealed risk stratification-specific signaling pathway activity characteristics (Figure 7B). The high-risk group showed significant activation of TGF Beta Signaling, Mitotic Spindle, and IL6 JAK STAT3 Signaling; while the low-risk group was characterized by Pancreas Beta Cells, Oxidative Phosphorylation, and KRAS Signaling DN. Correlation analysis between HMRS scores and these pathway activity scores further validated these findings (Figure 7C, *P* < 0.05).

To assess the clinical prognostic significance of key pathways, we selected six most significant signaling pathways for survival analysis, including Epithelial Mesenchymal Transition, Angiogenesis, Glycolysis, Apoptosis, Coagulation, and IL2 STAT5 Signaling. Kaplan-Meier analysis showed that high activity in these pathways was significantly associated with poorer overall survival (Figure 7D, all *P* < 0.001). Hazard ratio (HR) analysis further confirmed these outcomes and found that the role of Hedgehog signaling pathway as the sole protect prognostic factor (Figure 7E).

### 3.7 Analysis of somatic mutation spectrum and tumor heterogeneity

**FIGURE 8:**
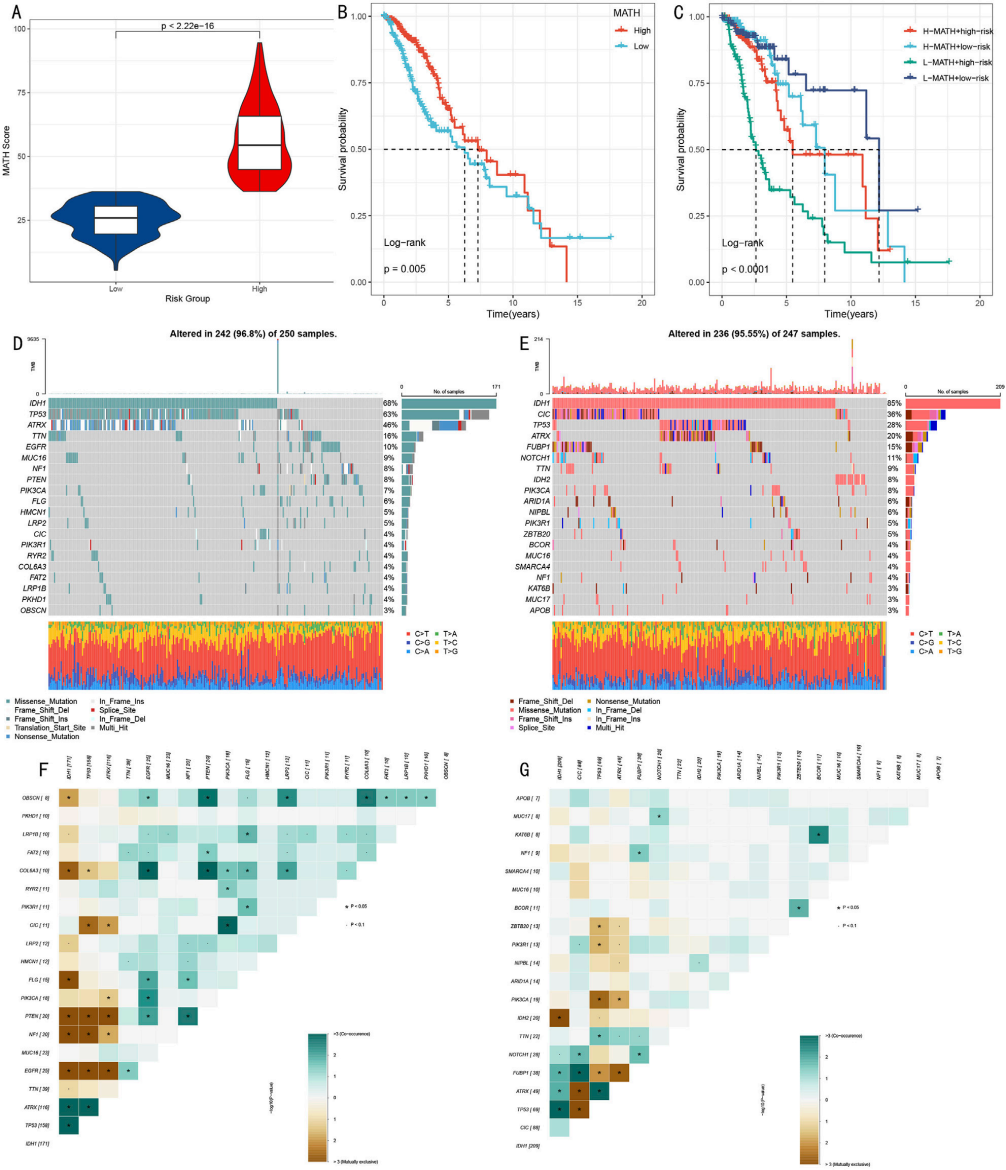
Multi-dimensional Analysis of Somatic Mutation Spectrum and Tumor Heterogeneity. **(A)** Box plot comparison of MATH scores between high and low score groups. **(B)** Kaplan-Meier survival analysis based on MATH scores. **(C)** Survival analysis combining MATH scores and risk scores. **(D, E)** Mutation landscape waterfall plots showing top 20 mutated genes in high-risk **(D)** and low-risk **(E)** groups. **(F, G)** Mutation gene co-occurrence/mutual exclusivity relationship heatmaps for high-risk **(F)** and low-risk **(G)** groups.

To deeply understand the genomic characteristics of LGG patients, we conducted systematic analysis of histone modification gene mutation patterns and tumor heterogeneity. Using MATH (Mutant-Allele Tumor Heterogeneity) scores to quantify intratumoral heterogeneity levels, results showed significant difference between high score group and low score group (Figure 8A, *p* < 0.001). Survival analysis based on MATH scores indicated that low MATH scores were significantly associated with poorer prognosis (Figure 8B, *p* = 0.005). Further analysis integrating MATH scores with HMRS risk stratification showed that the “low risk + low MATH” subgroup had the most favorable prognosis (Figure 8C, *p* < 0.001).

Through systematic analysis of mutation landscapes in high and low-risk groups (Figures 8D, E), we found: 1) TP53, as a key tumor suppressor gene, had a mutation frequency of 63% in the high-risk group, significantly higher than 28% in the low-risk group; 2) The transcription repressor CIC had a mutation frequency of 4% in the high-risk group compared to 36% in the low-risk group; 3) IDH1 mutations, associated with specific cytogenetic abnormalities and 1p/19q codeletion, showed mutation frequencies of 68% and 85% in high and low-risk groups respectively; 4) ATRX gene, involved in transcriptional regulation and chromatin remodeling, had mutation frequencies of 46% and 20% in high and low-risk groups respectively; 5) Additionally, characteristic mutations in the high-risk group included TTN (16%), while the low-risk group included FUBP1 (15%).

Mutation co-occurrence analysis (Figures 8F, G) revealed that in the high-risk group, TP53 mutations significantly co-occurred with IDH1 and ATRX. In the low-risk group, besides observing co-occurrence patterns of TP53, IDH1, and ATRX, significant mutation co-occurrence characteristics were also found between COL6A3 and PTEN.

### 3.8 Analysis of immune microenvironment characteristics and model associations

**FIGURE 9:**
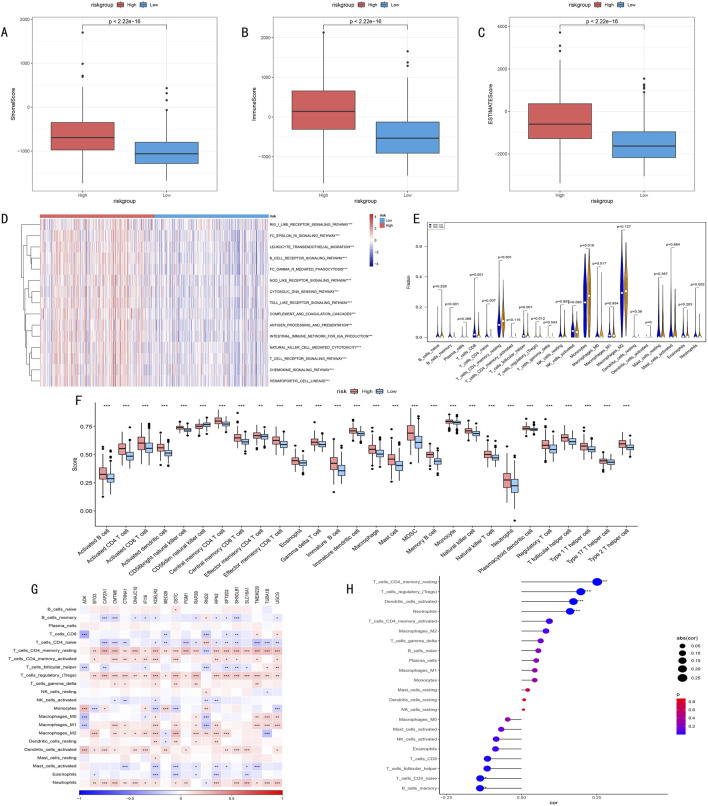
**(A–C)** ESTIMATE algorithm assessment of immune microenvironment differences between high and low-risk groups. **(A)** Stromal Score reflects tumor stromal components; **(B)** Immune Score quantifies immune cell infiltration levels; **(C)** ESTIMATE Score comprehensively characterizes tumor microenvironment features. **(D)** Heatmap of differential immune-related pathway activities between high and low-risk groups identified by ssGSEA algorithm, red indicates pathway upregulation, blue indicates pathway downregulation, color intensity represents degree of difference. **(E)** Violin plot of infiltration proportion differences of 22 immune cells between high and low-risk groups quantified by CIBERSORT algorithm. Shows distribution characteristics, density, and significant differences of each immune cell type. **(F)** Box plot of abundance differences of 28 characteristic gene-defined infiltrating immune cell types between high and low-risk groups. Box shows interquartile range, whiskers show 1.5 times interquartile range, outliers shown separately. **(G)** Correlation heatmap between risk score-related gene expression levels and various immune cell infiltration degrees. Red indicates positive correlation, blue indicates negative correlation, color intensity represents correlation strength. **(H)** Correlation scatter plot between risk scores and key immune cell infiltration levels. Point size represents absolute value of correlation coefficient, color indicates correlation direction and statistical significance.

We conducted multi-dimensional analysis of the immune microenvironment in high and low-risk groups. ESTIMATE algorithm assessment results showed (Figures 9A–C) that the high-risk group had significantly higher stromal scores, immune scores, and overall scores than the low-risk group (*p* < 0.001), suggesting more active immune responses and stromal components in the high-risk group.

Furthermore, ssGSEA algorithm analysis revealed all 15 significantly different immune-related pathways between high and low-risk groups (Figure 9D).

CIBERSORT algorithm analysis of immune cell infiltration characteristics showed (Figures 9E, F): 1) Memory CD4^+^ T cells were significantly higher in the high-risk group (*P* < 0.001); 2) Memory B cells, CD8^+^ T cells, and follicular helper T cells were more abundant in the low-risk group (*P* = 0.001); 3) Except for CD56dim NK cells, other immune cells generally showed higher expression levels in the high-risk group.

Correlation analysis between key gene expression and immune cell infiltration (Figure 9G) revealed that histone modification genes might participate in LGG progression by regulating immune cell infiltration. Correlation analysis between risk scores and immune cell infiltration (Figure 9H) indicated: 1) Significant positive correlations with memory CD4^+^ T cells, regulatory T cells, dendritic cells, and neutrophils; 2) Significant negative correlations with naive T cells and memory B cells. These findings suggest that HMRS can effectively quantify the immune status of LGG patients, reflecting significant immune landscape differences among patients with different risk levels.

### 3.9 Drug sensitivity analysis

**FIGURE 10:**
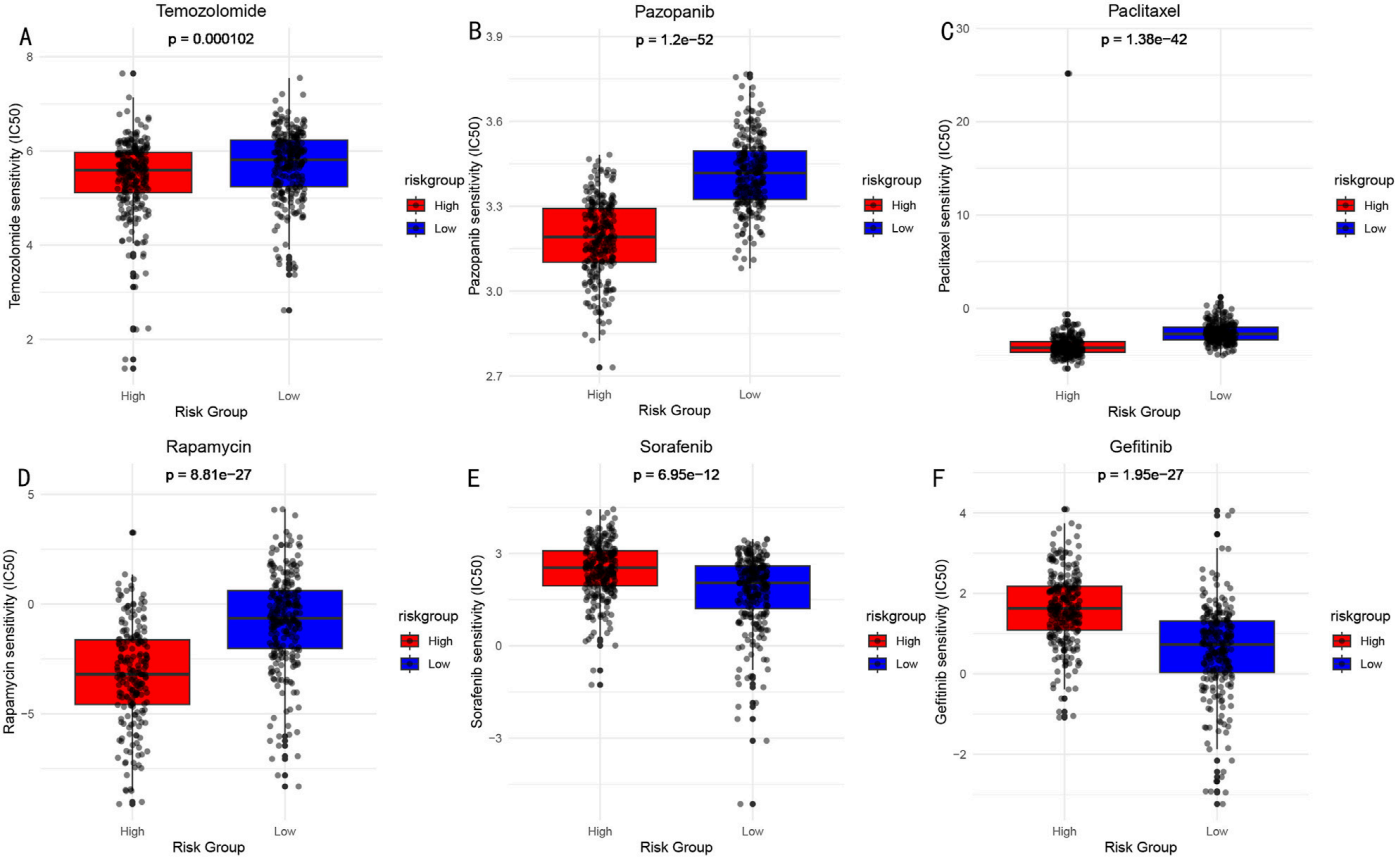
Drug Sensitivity Analysis (IC50 values) Between High and Low-risk Groups. **(A)** Temozolomide: DNA alkylating agent, widely used chemotherapy drug for brain glioma treatment. **(B)** Pazopanib: Multi-target tyrosine kinase inhibitor, used for treatment of various solid tumors. **(C)** Paclitaxel: Microtubule protein inhibitor, classic anti-tumor chemotherapy drug. **(D)** Rapamycin: mTOR pathway inhibitor, with immunosuppressive and anti-tumor effects. **(E)** Sorafenib: Multi-target tyrosine kinase inhibitor. **(F)** Gefitinib: EGFR tyrosine kinase inhibitor.

Based on the risk score model, we predicted sensitivity differences to common drugs between high and low-risk groups. Through comparison of IC50 values (Figure 10), significant response differences were found for the following drugs: Temozolomide (A), Pazopanib (B), Paclitaxel (C), Rapamycin (D), Sorafenib (E), and Gefitinib (F). These findings provide important references for risk stratification-based individualized medication.

### 3.10 HPA validation analysis

**FIGURE 11:**
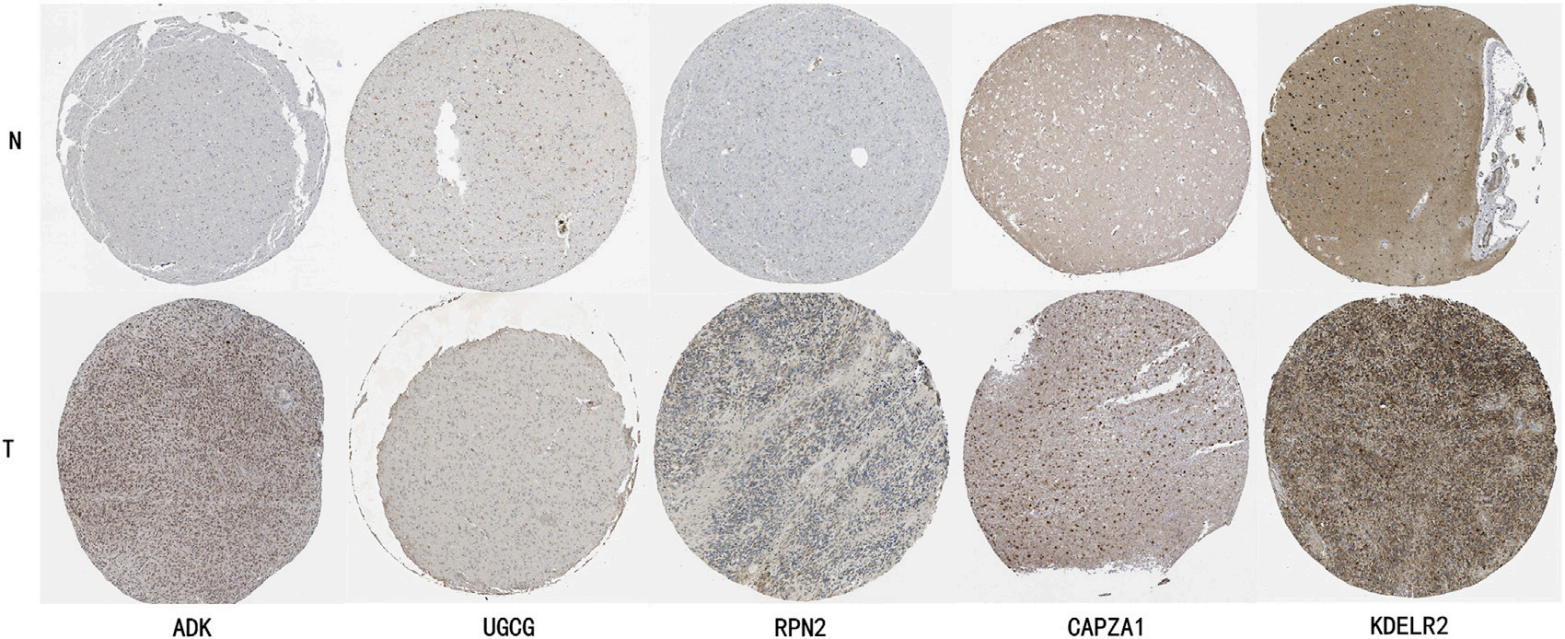
HPA Database Immunohistochemical Validation of Key Gene Expression Characteristics in LGG Tissue. ADK (Adenosine Kinase): Normal brain tissue shows light gray weak positive expression, while glioma tissue shows deep brown moderate to strong positive staining, mainly localized in cytoplasm. UGCG (UDP-Glucose Ceramide Glucosyltransferase): Normal brain tissue shows faint staining with almost no expression, glioma tissue shows obvious brown positive staining in cell membrane and cytoplasm. RPN2 (Ribophorin II): Normal brain tissue shows uniform light gray weak expression, glioma tissue shows uneven dark strong positive staining, mainly localized in endoplasmic reticulum. CAPZA1 (F-Actin-Capping Protein Subunit Alpha-1): Normal brain tissue shows weak expression, glioma tissue shows obvious brown moderate to strong positive staining, distributed in cytoplasm. KDELR2 (KDEL Endoplasmic Reticulum Protein Retention Receptor 2): Normal tissue shows light brown weak expression, glioma tissue shows obvious deep brown strong positive expression, mainly localized in Golgi apparatus.

To further validate the expression characteristics of key genes in the risk model, we selected five genes with the highest weight coefficients (ADK, UGCG, RPN2, CAPZA1, KDELR2) for HPA database immunohistochemical validation (Figure 11). Results showed that all these genes exhibited significantly high expression in LGG tissue.

### 3.11 Pan-cancer analysis

**FIGURE 12:**
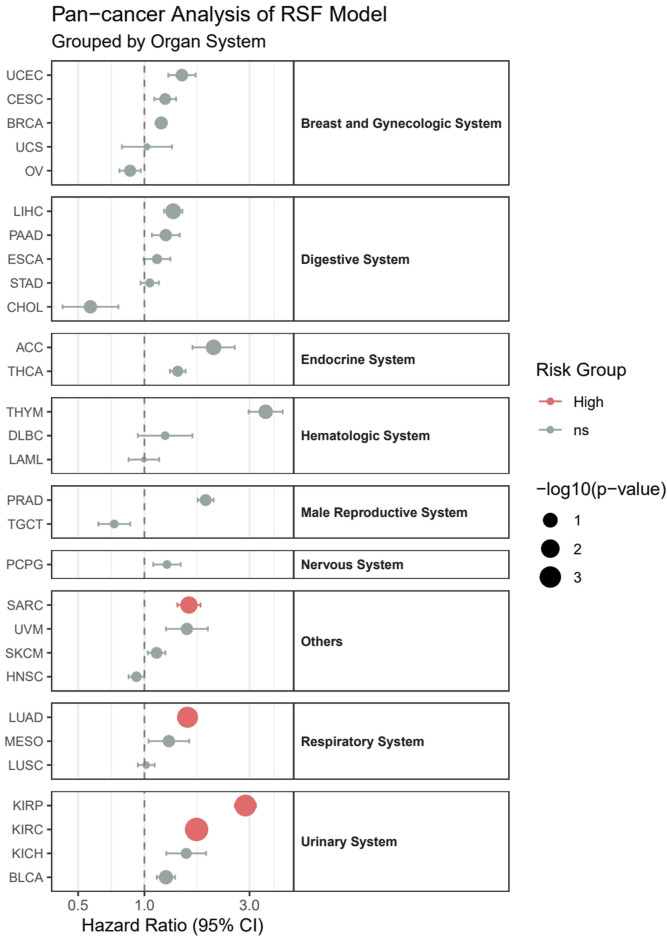
Pan-cancer analysis of survival outcomes across 29 cancer types stratified by organ systems.

Our pan-cancer analysis revealed distinct survival patterns across different cancer types and organ systems (Figure 12). Among the 29 cancer types analyzed, four cancer types demonstrated statistically significant associations with survival outcomes (*p* < 0.05). In the Urinary System, both Kidney Renal Clear Cell Carcinoma (KIRC, HR = 1.72, p = 0.00016) and Kidney Renal Papillary Cell Carcinoma (KIRP, HR = 2.87, *p* = 0.00077) showed significantly higher risk in the high-risk group. Within the Respiratory System, Lung Adenocarcinoma (LUAD) exhibited significantly poorer outcomes (HR = 1.57, *p* = 0.0014). Additionally, in the others category, Sarcoma (SARC) demonstrated significantly worse survival (HR = 1.59, *p* = 0.024).

Although not reaching statistical significance, several other cancer types showed notable trends:

1. Adrenocortical Carcinoma (ACC) displayed a strong tendency toward higher risk (HR = 2.06, *p* = 0.065).
2. Liver Hepatocellular Carcinoma (LIHC) showed a trend toward increased risk (HR = 1.35, *p* = 0.056).
3. Thymoma (THYM), despite having the highest hazard ratio (HR = 3.55), did not reach statistical significance (*p* = 0.116), possibly due to limited sample size (n = 121).

Notably, most cancer types (24/29) maintained hazard ratios above 1.0, suggesting a consistent trend toward worse outcomes in high-risk groups across different cancer types, although not all reached statistical significance. However, some cancer types, including Cholangiocarcinoma (CHOL), Head and Neck Squamous Cell Carcinoma (HNSC), Acute Myeloid Leukemia (LAML), Ovarian Serous Cystadenocarcinoma (OV), and Testicular Germ Cell Tumors (TGCT), showed hazard ratios below 1.0, indicating potentially better outcomes in the high-risk group, though these associations were not statistically significant.

## 4 Discussion

### 4.1 Significance of histone modification heterogeneity

In this study, we first revealed the cellular heterogeneity characteristics of histone modifications in LGG through multi-omics analysis and developed a prognostic prediction model with clinical application prospects. Single-cell level analysis showed that tumor cells and oligodendrocytes exhibited higher levels of histone modifications, which is consistent with previous reports on the key role of histone modifications in glioma stem cell maintenance (Liu et al., 2024; Zhu et al., 2024; Liu et al., 2023). High levels of histone modifications may promote tumor cell proliferation and stemness maintenance through precise regulation of gene expression networks (Sharma et al., 2023; Shen et al., 2020), and this epigenetic level cellular heterogeneity may be one of the important reasons leading to LGG treatment resistance and recurrence. Based on this finding, we constructed the HMRS prognostic model, which demonstrated excellent predictive performance across multiple independent cohorts. The model’s prediction accuracy was further improved after integration with clinical features. The advantages of the HMRS model lie in its robustness, practicality, and individualization characteristics, providing a new tool for LGG patients’ prognostic assessment and treatment decision-making. These findings not only deepen our understanding of LGG epigenetic heterogeneity (Chaligne et al., 2021) but also provide an operational prognostic assessment method for clinical practice.

### 4.2 Advantages of the HMRS model

Compared with currently widely used prognostic assessment models, the HMRS model shows unique advantages. Traditional prognostic assessments mainly rely on WHO grading, IDH mutation status, and 1p/19q codeletion as molecular markers. Although these indicators have important prognostic implications, they often cannot fully reflect the molecular heterogeneity and dynamic evolution characteristics of tumors (Eckel-Passow et al., 2015). Recently developed radiomics-based prediction models, such as those combining MRI imaging features with machine learning (Li et al., 2021b), although advantageous in non-invasive assessment, still need improvement in prediction accuracy and stability. In contrast, our HMRS model not only integrates histone modifications as an important epigenetic feature but also ensures its predictive reliability through multi-center validation. Notably, the HMRS model can reflect the epigenetic heterogeneity of tumor cells, giving it potential advantages in predicting treatment response and guiding individualized treatment. Furthermore, the inclusion of clinical features makes it more easily applicable in actual clinical work, an advantage not possessed by other single molecular markers or complex models.

### 4.3 Molecular mechanisms and pathway analysis

Through functional enrichment analysis of high and low-risk groups, we deeply revealed the molecular mechanisms behind HMRS stratification. The study found multiple key signaling pathways significantly activated in the high-risk group, most notably the TGF-β and IL6-JAK-STAT3 signaling pathways. TGF-β signaling pathway activation may enhance tumor cell invasion and metastasis capabilities through inducing epithelial-mesenchymal transition (EMT) (Meng et al., 2016), while IL6-JAK-STAT3 pathway activation suggests the important role of inflammatory microenvironment in LGG progression, with this chronic inflammatory state potentially promoting tumor malignant progression through multiple mechanisms (Zhang et al., 2023). Meanwhile, we observed significant differential expression of metabolism-related pathways, reflecting the metabolic heterogeneity developed by tumor cells to adapt to malignant proliferation. These findings not only explain the intrinsic mechanisms of prognostic differences between different risk stratification patients at the molecular level but also provide theoretical basis for developing new therapeutic strategies, particularly targeted therapy against these abnormally activated pathways as potential treatment options for high-risk patients. Importantly, the interactions between these pathways form a complex regulatory network, suggesting the need to consider multi-target combined intervention when developing treatment strategies.

Compared to previous studies, the uniqueness of our research lies in being the first to systematically reveal the role of histone modification-mediated signaling pathway networks in LGG progression. Previous studies mainly focused on single pathways, such as IDH mutation-mediated metabolic reprogramming reported by Wang et al. (2023), or Guo et al. (2023)’s discovery of Notch signaling regulation in glial cell development and tumorigenesis. Through integrative analysis, our study not only confirmed the importance of these known pathways but also discovered complex regulatory relationships between them. Notably, we found that the TGF-β signaling pathway may influence global epigenetic states by regulating histone-modifying enzyme expression, a finding that echoes Mao et al. (2020)’s recent discovery in glioma but is the first report in LGG. Furthermore, our study first revealed potential connections between inflammatory pathways and metabolic reprogramming, providing new perspectives for understanding LGG heterogeneity and theoretical basis for developing multi-target combination therapy strategies.

### 4.4 Immune microenvironment characteristics

Further pathway analysis revealed comprehensive activation of both innate and adaptive immune response networks in high-risk patients. The enrichment of pattern recognition receptor pathways (RIG-I-like, NOD-like, and Toll-like receptor signaling) indicates heightened innate immune surveillance, potentially triggered by tumor-derived danger signals. The concurrent activation of B cell receptor signaling pathway, T cell receptor signaling pathway, and natural killer cell-mediated cytotoxicity suggests broad engagement of adaptive immune responses. The upregulation of leukocyte transendothelial migration and chemokine signaling pathways points to active immune cell trafficking within the tumor microenvironment. Additionally, the enrichment of antigen processing and presentation pathways, along with the complement and coagulation cascades, indicates robust immune recognition and response mechanisms. However, despite this extensive immune activation, the apparent ineffectiveness in tumor control suggests potential immune dysfunction or suppression. The enhanced FC gamma R-mediated phagocytosis pathway might reflect increased clearance of antibody-coated tumor cells, yet the overall immune response appears insufficient to prevent disease progression in high-risk patients. These pathway alterations, combined with the observed immune cell composition changes, paint a picture of a complex but potentially dysfunctional immune response that may contribute to tumor progression (Giannone et al., 2020; Kaur et al., 2022).

Then, our study revealed close associations between HMRS risk stratification and tumor immune microenvironment through multi-dimensional analysis. Through ESTIMATE algorithm analysis, we found that high-risk group patients showed significantly elevated immune scores and stromal scores, suggesting the existence of a more complex immune regulatory network. Notably, we observed significantly increased memory CD4^+^ T cells in the high-risk group, while other immune cells such as memory B cells, CD8^+^ T cells, and follicular helper T cells showed relative deficiency. This immune cell component remodeling may reflect the establishment of tumor immune escape mechanisms. Meanwhile, the activation of multiple immune-related signaling pathways, including RIG-I-like receptor signaling pathway, B cell receptor signaling pathway, and Toll-like receptor signaling pathway, further supports the key role of immune microenvironment in LGG progression. These findings not only deepen our understanding of the LGG immune microenvironment but more importantly provide new perspectives for developing immunotherapy strategies. Particularly for high-risk patients, rebuilding effective anti-tumor immune responses, such as enhancing memory CD8^+^ T cell function or regulating specific immune pathway activity, may become important strategies for improving treatment efficacy. Additionally, these immune characteristic differences suggest the need to consider individualized immune microenvironment differences when designing treatment plans, potentially requiring treatment strategy adjustments based on patients’ immune status, including whether to combine immune checkpoint inhibitors and other immunotherapy approaches.

### 4.5 Clinical applications and drug sensitivity

In terms of drug applications, temozolomide, the standard first-line treatment for LGG, showed significant therapeutic differences between high-risk and low-risk groups (Tomar et al., 2021), providing direct guidance for clinical medication decisions. Meanwhile, we observed that the high-risk group demonstrated good sensitivity to certain multi-target tyrosine kinase inhibitors such as pazopanib (Miyamoto et al., 2018), which is consistent with the abnormal pathway activation patterns we previously identified. Interestingly, high-risk group patients also showed increased sensitivity to traditional chemotherapy drugs like paclitaxel (Alqahtani and Aleanizy, 2019), suggesting that cell cycle regulation may be a crucial factor affecting drug response. Individualized treatment strategies not only hold promise for improving therapeutic outcomes but may also reduce unnecessary drug toxicity, thereby enhancing patients’ quality of life.

### 4.6 Pan-cancer applications and developmental biology perspective

Our pan-cancer analysis demonstrates that the model initially developed for LGG exhibits predictive value across multiple cancer types, achieving statistical significance particularly in kidney cancers (KIRC, KIRP) and lung adenocarcinoma (LUAD). This cross-organ system predictive capacity can be understood through the lens of evolutionary conservation in developmental biology. Although these tissues originate from different germ layers (neuroectoderm vs. mesoderm), they share several key regulatory networks during embryonic development.

Notably, kidney development involves complex mesenchymal-epithelial transition (MET) and neuroectoderm-mesoderm interactions, which share many similarities with cell fate determination mechanisms in neural system development. For instance, WT1 and the PAX gene family play crucial roles in both kidney and neural system development (Discenza et al., 2003; Ochi et al., 2022). Similarly, lung development requires precise EMT and complex cell lineage determination, sharing multiple regulatory pathways with neural crest cell migration and differentiation mechanisms (Jolly et al., 2018). These common developmental features may explain why epigenetic markers derived from neural system tumors demonstrate significant predictive value in these cancer types.

From an evolutionary developmental perspective, this conservation of predictive features reflects the shared pluripotent state and fundamental regulatory mechanisms of different tissues during early embryonic development. Despite subsequent divergence in differentiation pathways, the basic epigenetic regulatory networks remain highly conserved throughout evolution.

### 4.7 Limitations and future directions

Although our study has made several important discoveries in LGG histone modifications and prognostic prediction, there are still some limitations that need to be addressed in future research. The primary limitation is the lack of prospective clinical validation; although our model has demonstrated good predictive performance across multiple independent cohorts, its performance in real clinical settings still needs to be verified through prospective studies. Secondly, the functional mechanisms of key genes still need more experimental data support, particularly *in vivo* and *in vitro* functional experiments will help to deeply understand these genes’ specific roles in LGG progression. Additionally, while drug sensitivity analysis provides important clues for individualized treatment, these predicted results still need to be validated through standardized clinical trials for accuracy and reliability. Finally, the dynamic nature of epigenetic modifications suggests that longitudinal sampling might provide additional insights not captured in our current cross-sectional analysis. Based on these limitations, we have planned several important future research directions: first, we will conduct multi-center prospective clinical studies to systematically evaluate the clinical application value of the HMRS model; second, through in-depth molecular biology experiments, we will elucidate the regulatory mechanisms of key genes and their roles in tumor progression; third, we will explore individualized combination treatment strategies based on risk stratification, particularly in optimizing combinations of immunotherapy and targeted therapy; finally, we plan to integrate multi-modal data such as radiomics and metabolomics to develop more comprehensive and precise prediction models. These studies will help to further improve the diagnosis and treatment level of LGG patients, ultimately achieving better clinical outcomes.

## 5 Conclusion

This study establishes a novel HMRS for LGG through comprehensive multi-omics analysis. The model demonstrates robust predictive performance across multiple independent cohorts and reveals distinct molecular and immune characteristics between risk groups. High-risk tumors show activation of specific signaling pathways (particularly TGF-β and IL6-JAK-STAT3), distinct mutation profiles, and unique immune cell infiltration patterns. The model also provides valuable insights into drug sensitivity, suggesting potential therapeutic strategies for different risk groups. Furthermore, pan-cancer analysis indicates the model’s broader applicability across multiple cancer types, particularly in kidney and lung cancers. While the model shows promise for clinical application in personalized treatment planning, future prospective studies are needed to validate its clinical utility. This integrated approach advances our understanding of LGG biology and offers a framework for improving patient care through molecular-based risk assessment and treatment selection.

## Data Availability

The data presented in the study are deposited in the following repositories: 1. Single-cell RNA sequencing data are deposited in the Gene Expression Omnibus (GEO) database, accession number GSE182109 (https://www.ncbi.nlm.nih.gov/geo/). 2. Bulk RNA sequencing data are accessible through The Cancer Genome Atlas (TCGA-LGG) dataset and the Genotype-Tissue Expression (GTEx) project. 3. Validation datasets are available in the Chinese Glioma Genome Atlas (CGGA) database (https://www.cgga.org.cn/). All these datasets are publicly available through their respective repositories.
